# The Placenta as a Diagnostic Specimen in Disseminated Tuberculosis of Pregnancy presenting as Acute Respiratory Distress Syndrome – A case report

**DOI:** 10.1016/j.idcr.2026.e02810

**Published:** 2026-10-24

**Authors:** Bhvika Zutshi, Sandeep Garg, Praveen Bharti, Amit Kumar, Annesha Chakraborty, Priyadarshi Meena

**Affiliations:** Department of General Medicine, Maulana Azad Medical College and Associated Hospitals, New Delhi, India

**Keywords:** Disseminated tuberculosis, Pregnancy, Acute respiratory distress syndrome, Tuberculoma, Placental tuberculosis, CBNAAT

## Abstract

Tuberculosis in pregnancy is characteristically paucibacillary, and conventional maternal specimens frequently fail to yield a diagnosis. The placenta is available at every delivery yet is seldom submitted for molecular testing. A 22-year-old unregistered primigravida at 16 weeks of gestation presented with fever, dry cough and progressive dyspnea meeting criteria for the acute respiratory distress syndrome (ARDS). Tuberculin testing was anergic, sputum was unobtainable, bronchoalveolar lavage was precluded by hypoxemia and obstetric risk, and virological, bacteriological, autoimmune and fungal evaluation was unrevealing. Empirical antitubercular therapy begun in the third week was withdrawn for hepatotoxicity. Recrudescent fever with headache yielded a lymphocytic, low-glucose, high-protein cerebrospinal fluid consistent with probable tuberculous meningitis (Marais score 12) despite negative cerebrospinal-fluid nucleic acid amplification (CBNAAT) testing. After spontaneous preterm delivery and neonatal death, imaging demonstrated pulmonary tree-in-bud nodularity and cerebral, cerebellar, and brainstem tuberculomas. Placental histopathology showed caseating granulomatous villitis with Langhans giant cells; Ziehl-Neelsen, Fite, Gomori methenamine silver and Warthin-Starry stains all were negative. CBNAAT of fresh placental tissue detected rifampicin-sensitive Mycobacterium tuberculosis and mycobacterial blood culture subsequently demonstrated bacillemia. The mother improved on anti-tubercular therapy on follow-up. In this review of reported placental tuberculosis, molecular testing consistently outperformed acid-fast staining, and histological appearances were frequently nongranulomatous. The placenta should be submitted fresh for NAAT wherever tuberculosis is suspected in pregnancy, since it can establish the molecular diagnosis and provide rifampicin sensitivity when other maternal specimens have been inconclusive, and satisfies the Cantwell criterion that guides neonatal evaluation.

## Introduction

Tuberculosis disproportionately affects women of reproductive age, and pregnancy, both increases susceptibility and obscures recognition [1], [2]. The immunological reorientation of gestation attenuates Th1 responses and mycobacterium tuberculosis-specific interferon γ production, producing a paucibacillary, immune-tolerant phenotype where not just the tuberculin testing, but even the molecular and bacteriological assays lose sensitivity [3], [4], [5], [6]. Tuberculosis accounts for < 4% of acute respiratory distress syndrome admissions even in dedicated respiratory intensive care units in high burden tertiary settings [7]. Compounding this, sputum induction often fails, bronchoscopy deferred for obstetric risk and hypoxemia, and imaging delayed for fetal protection. The result is a diagnostic impasse in which clinical suspicion outruns microbiological proof.

The placenta occupies a unique and underexploited position in this impasse. It is an organ perfused directly by maternal blood; anatomical site at which hematogenous maternal infection is filtered and deposited, and uniquely among deep tissues, it is discarded or, at best submitted for histopathology alone. Morphology is imperfect: granulomas may be absent, and acid-fast staining of placental tissue is insensitive [8], [9], [10].

We report a case in which every maternal airway and T-cell based investigation failed, and in which CBNAAT of fresh placental tissue established the diagnosis and rifampicin sensitivity. This case is being used to frame a focused review of diagnostic performance of placental specimens across assay types and to position for the routine molecular interrogation of the placental specimen when tuberculosis is suspected in pregnancy.

## Case presentation

A 22-year-old unregistered primigravida at 16 weeks and 2 days of gestation was referred with subacute fever, dry cough of 15–20 days, and breathlessness progressing over 3–4 days. She had no prior tuberculosis, no contact history, no comorbidity, and no BCG scar on left deltoid region (weight 52 kg, BMI 18.6 kg/m^2^). On arrival she was tachypneic (rate 45 breaths/min) with spO2 90% under room air, temperature 38.3 ^º^C and bilateral crackles. Arterial blood gas analysis on room air had a pH of 7.46, PaO_2_ 59.4 mm Hg PaCO_2_ 20.6 mm Hg and HCO_3_^-^ 18.2 mmol/L: acute respiratory alkalosis superimposed on physiological alkalosis of pregnancy with an alveolar-arterial gradient of approximately 60 mm Hg. Saturation later fell to 75%, requiring continuous positive airway pressure at 6 cm H2O, on which the PaO2/FiO2 ratio was 300. With the bilateral airspace opacities and normal bedside echocardiography **(**Fig. 1**)**, her presentation satisfied both the Berlin definition [11] and the non-intubated category of 2024 Global definition of ARDS [12]**.** The diagnostic sequence is summarised in Table 1. Two sequential multiplex viral panels, routine blood, urine and induced sputum cultures, HIV serology, an autoimmune screen, fungal studies and serology for leptospirosis, scrub typhus, syphilis, hepatitis A and E were negative. Bronchoalveolar lavage was deferred due to obstetric risk and hypoxemia. High-resolution computed tomography done in the third week **(**with high-risk informed consent, Fig. 2**)** showed bilateral ground-glass attenuation with centrilobular nodularity, prompting empirical antitubercular therapy according to National Tuberculosis Elimination Programme (NTEP) guidelines (weight-based HRZE with pyridoxine). Within 7 days, this precipitated hepatotoxicity (AST/ALT 423/346 IU/L; total bilirubin 2.6 mg/dL), meeting Hy’s law, hence the regimen was withdrawn [11].

**Fig. 1:**
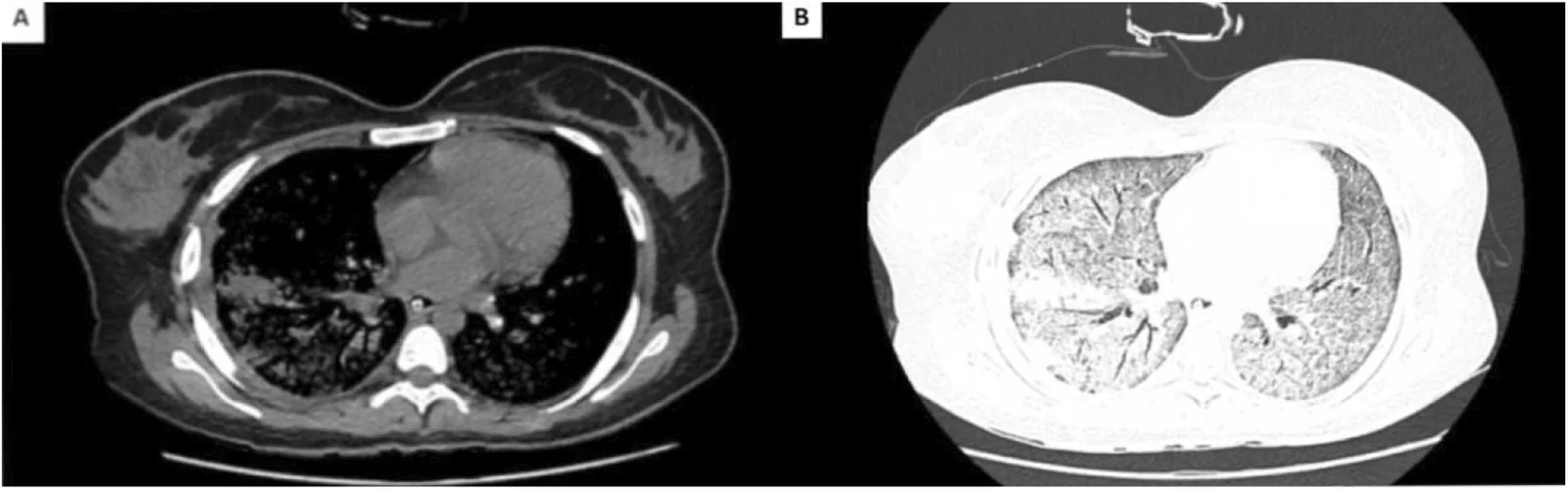
Axial sections of high-resolution computed tomography (HRCT) of the chest (third week, performed with abdominal shielding) showing bilateral, symmetric discrete-to-confluent centrilobular nodular opacities with ground-glass attenuation, dependent consolidation, and air bronchograms, without mediastinal lymphadenopathy. **[A, B]**.

**Table 1:** Timeline

**Time point**	**Clinical evolution**	**Key investigations/findings**	**Management**
Preadmission	Fever, dry cough, progressive dyspnoea; treated at a local hospital.	Outside CXR - bilateral bibasal and peripheral confluent symmetric opacities.	O₂, meropenem, paracetamol, fluids; referred.
Day 1 (admission, 16w+2d)	Rest dyspnoea, SpO₂ 90% under room air, febrile, tachypnoeic.	ABG PaO₂ 59.4, PaO₂/FiO₂ ≈ 283; CXR bilateral confluent opacities; USG chest - no effusion.	CPAP 6 cm H₂O; meropenem, teicoplanin, azithromycin, oseltamivir; IV methylprednisolone.
Week 1	Persistent fever, saturation (90→81% under room air).	Echo EF - 60%, proBNP normal; D-dimer 1887 ng/mL, Doppler - no evidence of deep venous thrombosis; viral panel negative; cultures sterile.	Continued support and antimicrobials.
Week 2	Fever persists; slight symptomatic improvement.	HIV non-reactive; ANA/dsDNA and autoimmune-hepatitis panels negative; dengue IgM reactive (deemed a confounder), IgG negative.	Steroid taper planned; supportive care.
Week 3	Non-resolving illness.	Immunoglobulins normal; fungal work-up negative; CD4 217/µL; HRCT - ground glass opacities, dependent consolidation, centrilobular nodularity, no lymphadenopathy.	Empirical ATT started (high teratogenic-risk consent).
Week 4	Gradual respiratory improvement; ATT-related hepatic flare.	AST/ALT peak 423/346 IU/L; second viral panel negative.	ATT withheld; cotrimoxazole PCP prophylaxis; O₂ via nasal prongs; level-II obstetric scan normal.
Weeks 5–6	Afebrile, SpO₂ 95% under room air, respiratory rate 30; hacking cough persists.	Inflammatory markers and transaminases normalising; CD4 recovers to 325/µL; serial CXRs demonstrating regression of basal opacities.	Oral steroid taper; supportive care; shifted out of critical care.
Relapse (postpartum ward)	Recurrent fever with intractable headache and vomiting; no focal deficit or meningism.	CSF: 25 cells (65% lymphocytes), glucose 30, protein 109, ADA 8.9; AFB/CBNAAT/India-ink negative.	ATT reinstituted with dexamethasone and mannitol.
Delivery outcome & discharge (6–8 weeks)	Spontaneous preterm vaginal delivery at 23w; extremely-low birth-weight (685 gm), neonate died despite intubation. Mother survived.	CECT chest - tree-in-bud nodularity; CECT brain - multiple cerebral/cerebellar/brainstem tuberculomas; placenta - granulomatous villitis with Langhans giant cells. Fresh placental tissue GeneXpert positive.	Continued ATT and neuro-protective measures; obstetric care.
Follow up (~4 weeks and beyond post discharge)	Significant improvement in general condition.	LJ media blood culture for MTB reported as positive, Rif and Inh sensitive; MRI Brain later showed partial resolution of granulomas.	ATT continued with monthly LFT monitoring.

**Fig. 2:**
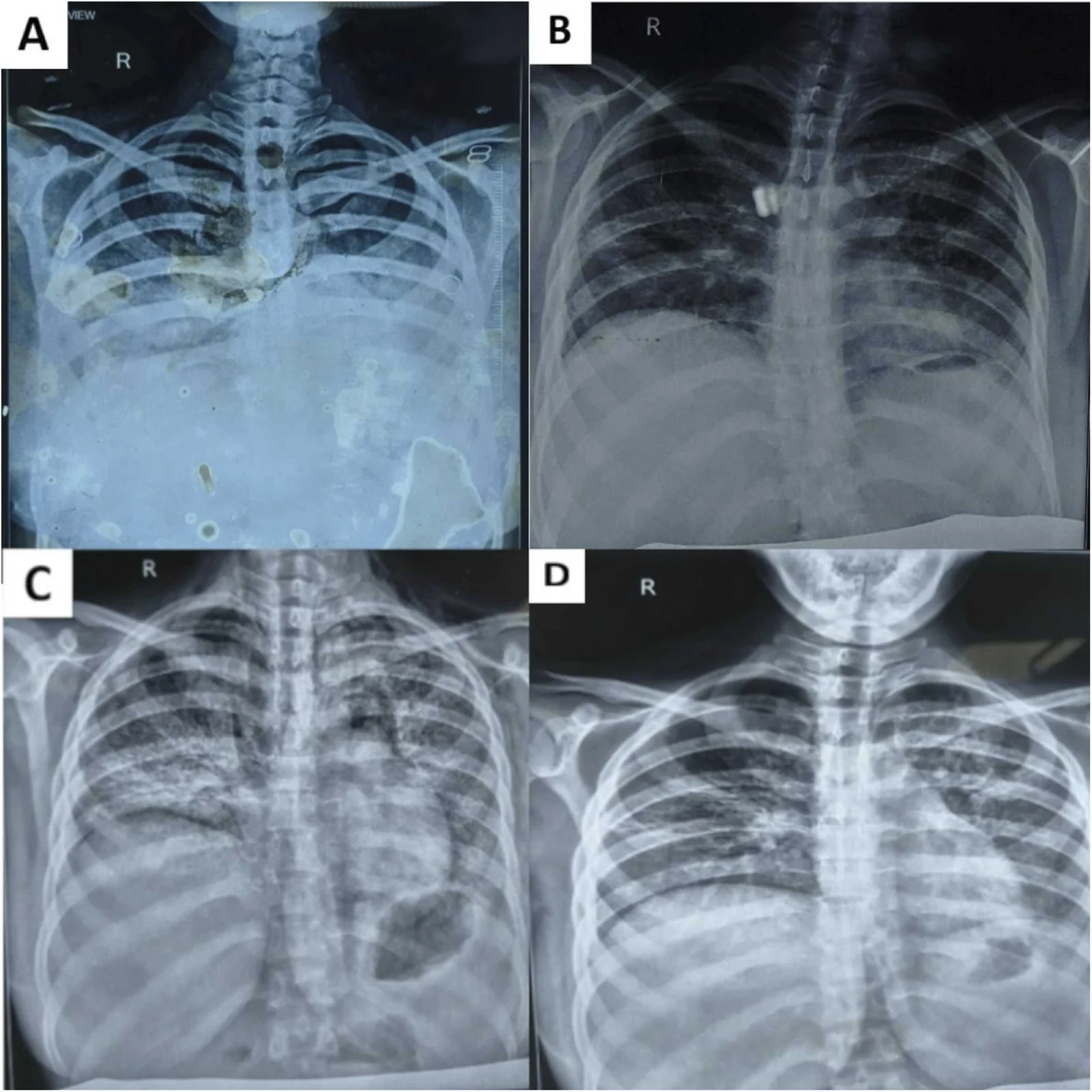
Sequential chest radiographs demonstrating progressive regression of bilateral basal opacities during supportive therapy. **[A]** Admission; **[B]** Week 3; **[C]** Week 5; **[D]** Discharge.

Following clinical improvement and transfer from critical care, fever recurred with intractable pan-cranial headache. Cerebrospinal fluid showed 25 cells/µL (65% lymphocytes), glucose 30 mg/dL, protein 109 mg/dL and adenosine deaminase 8.9 U/L, with a negative smear, cryptococcal antigen, acid-fast stain, CBNAAT and culture, amounting to probable tuberculous meningitis by the Marais criteria [12] **(score 12).** Antitubercular therapy was reinstituted with dexamethasone and mannitol, and mycobacterial cultures sent as well.

Spontaneous preterm delivery occurred at 23 weeks, and the neonate (685 g birth weight) was intubated for respiratory distress, however died. The fresh placental tissue was submitted for histopathology and molecular testing. Postpartum imaging showed pulmonary tree-in-bud nodularity **(**Fig. 3**)** and multiple ring enhancing diffuse cerebral, cerebellar and brainstem lesions with perilesional edema and no identifiable scolices, consistent with tuberculomas **(**Fig. 4**).**

**Fig. 3:**
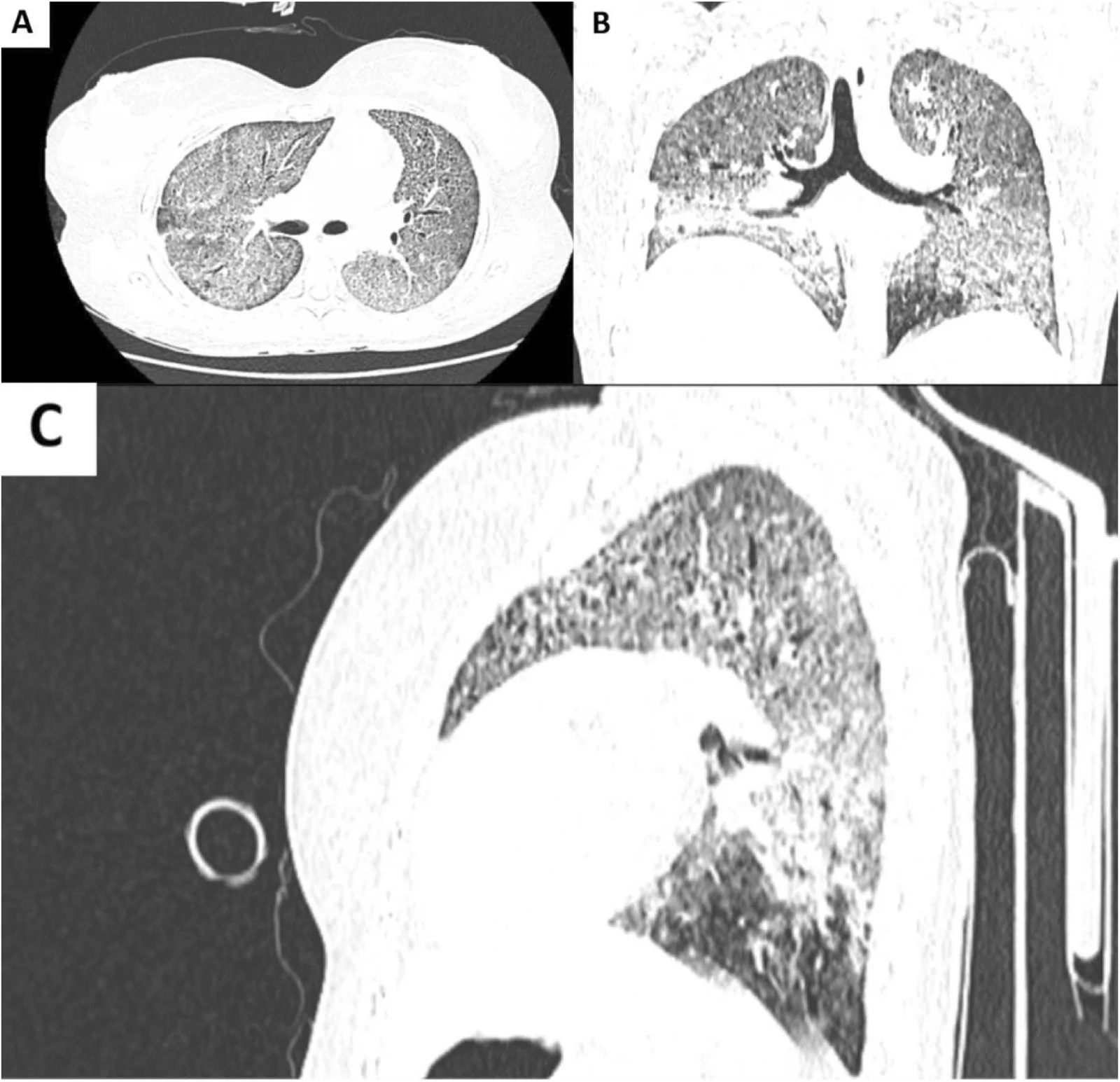
Postpartum contrast-enhanced computed tomography (CECT) of the chest demonstrating discrete-to-confluent centrilobular nodules in a tree-in-bud configuration without mediastinal lymphadenopathy, consistent with active endobronchial tuberculous spread. **[A–C]**.

**Fig. 4:**
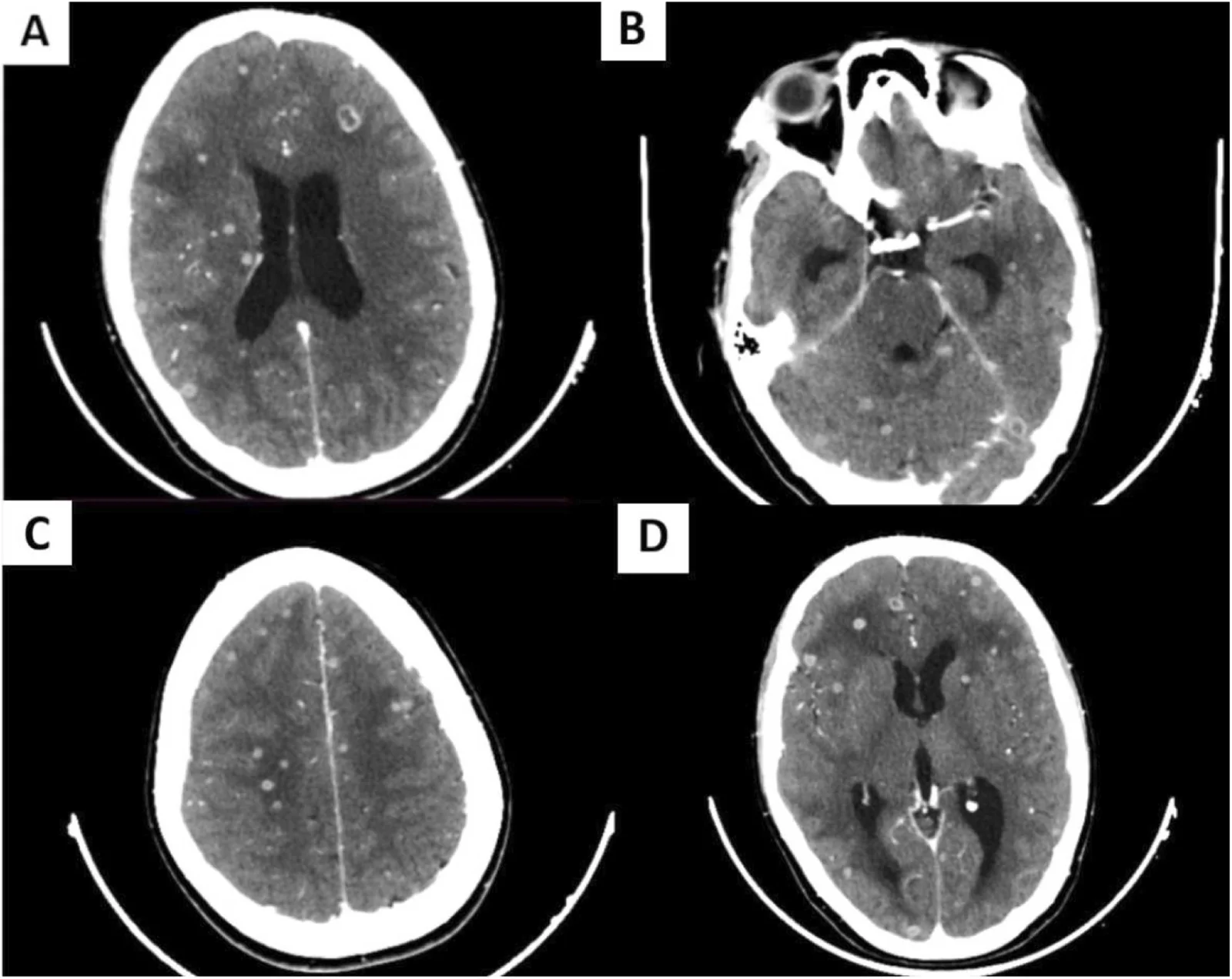
Postpartum CECT brain demonstrating multiple variably-sized, discrete-to-conglomerate, rim-enhancing nodular lesions in both cerebral and cerebellar hemispheres and the brainstem, with perilesional oedema. No scolex is identifiable. Findings are consistent with cerebral tuberculomas. **[A–D]**.

Placental histopathology **(**Fig. 5**)** demonstrated caseating granulomatous villitis with epithelioid granulomas and Langhans type multinucleated giant cells. Ziehl-Neelsen, Fite, Gomori methenamine and Warthin-Starry stains were negative, and repeat syphilis serology was also non-reactive, excluding other principal causes of granulomatous villitis. CBNAAT performed on fresh placental tissue detected M. tuberculosis with no rifampicin resistance. Mycobacterial blood culture, drawn at the reinstitution of therapy, was reported at one-month followup, grew rifampicin and isoniazid sensitive M. tuberculosis, confirming the bacillemia and hematogenous dissemination.

**Fig. 5:**
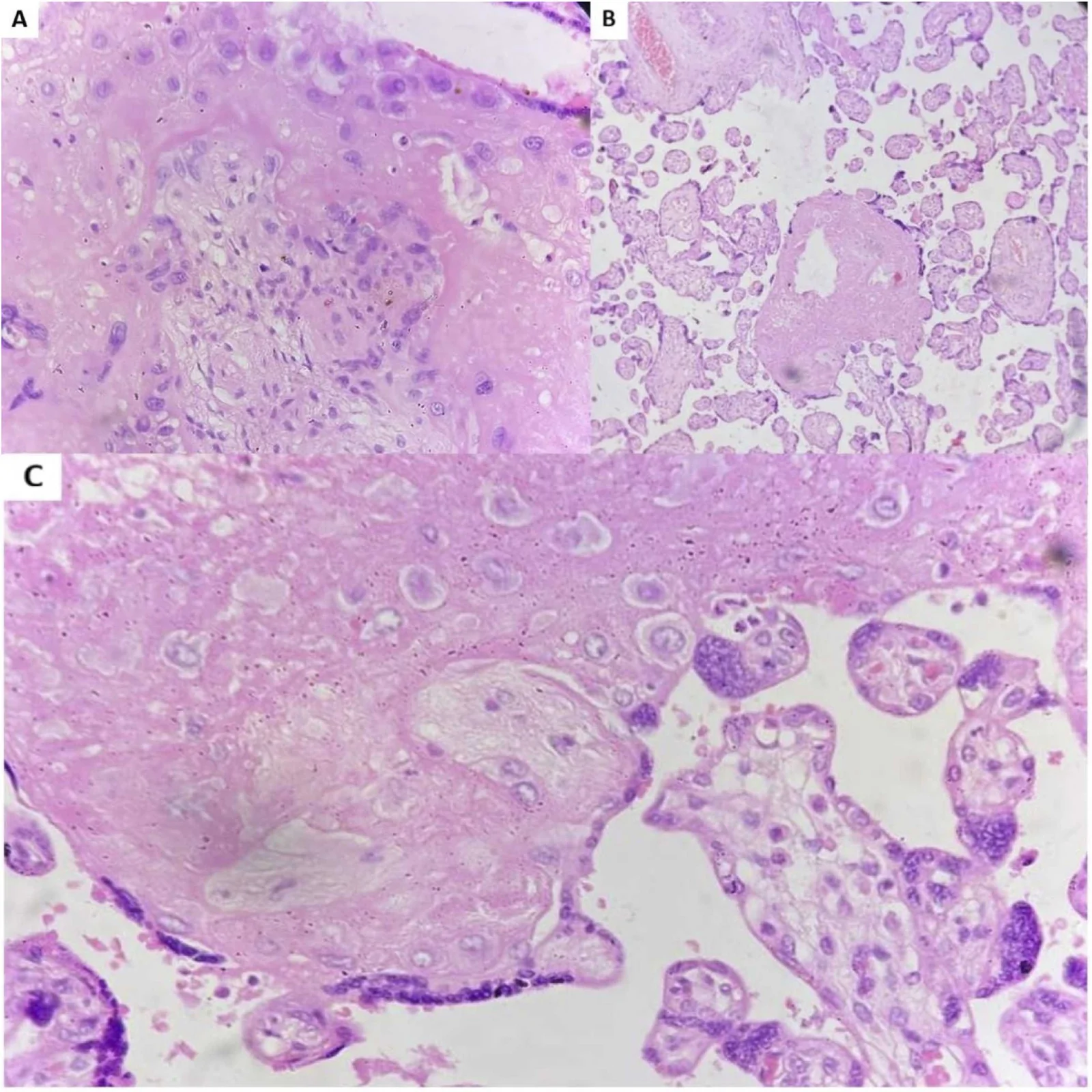
Haematoxylin and eosin stained sections of placenta **[A-C]** suggestive of acute chorioamnionitis with villous inflammation and large giant cells and epithelioid granulomas. **[10X]**. Ziehl–Neelsen, Fite, GMS.

The patient was discharged on 2-month intensive phase of HRZE, followed by 10–16 month continuation phase of HRE, with tapering dexamethasone, pyridoxine supplementation, and monthly liver function monitoring and was duly notified to NTEP. At 3 months, magnetic resonance imaging showed partial resolution of intracranial lesions, confirming treatment response and arguing against other differentials of intracranial ring-enhancing lesions.

## Discussion

The instructive feature of this case is not just the severity of the illness but the anatomical distribution of the diagnostic yield. Every specimen sampling of the airway and the T-cell memory was negative or unobtainable, and both the specimens derived from the blood compartment were diagnostic **(**Table 1**).** Pregnancy attenuates the Th1 responses that generate the expectorated bacilli and tuberculin reactivity [5], [6], whereas maternal bacillemia continues to deposit organisms in the intervillous spaces irrespective of the host immunophenotype. Where the airway cannot be sampled in a gravid woman, the blood compartment, the placenta being its most accessible proxy, should be evaluated early molecularly.

Placental involvement in maternal tuberculosis is neither rare nor reliably granulomatous, and the published experience shows a consistent hierarchy of assay performance **(**Table 2**).** In the largest dedicated series, nineteen placentas from pregnant women with tuberculosis were examined at a single referral centre; molecular testing was positive in all, whereas the classical morphology and acid-fast staining were considerably less reliable, and 3 of the 9 surviving infants subsequently developed congenital tuberculosis [8]**.** Two placentas reported by Abramowsky and colleagues showed no granuloma at all, exhibiting instead an acute neutrophilic villitis and intervillitis in which bacilli were demonstrable only on fluorescent staining [9]; a further case manifested as acute chorioamnionitis [10]. Morphology alone therefore cannot be relied upon to raise or exclude the diagnosis.

**Table 2:** Reported diagnostic performance of placental and tissue specimens in tuberculosis,by assay type

**Study**	**Specimen**	**Assay(s)**	**Diagnostic Yield**	**Impact on management**
Hu et al., 2023	Placenta (19), single-centre series	H&E, acid-fast stain, molecular (PCR)	PCR positive in all 19; morphology and staining less consistent	Confirmed maternal diagnosis; directed neonatal follow-up — 3/9 surviving infants developed congenital TB
Abramowsk y et al., 2012	Placenta (2)	H&E, auramine O fluorescence	Granulomas absent; acute neutrophilic villitis/intervillitis; bacilli seen on fluorescent stain	Demonstrates that nongranulomatous morphology does not exclude placental TB
Taweevisit et al., 2015	Placenta (1) with literature review	H&E, acid-fast stain	Acute chorioamnionitis pattern rather than granulomatous placentitis	Widens the histological differential; supports adjunctive testing
Njau et al., 2019	Archival FFPE tissue (80), extrapulmonary sites	Xpert MTB/RIF vs Ziehl– Neelsen (histology reference)	Xpert 53.2% (95% CI 41.6–64.9) vs ZN 20.3% (95% CI 12–30.8); p = 0.002	Molecular testing markedly outperforms staining on fixed tissue
Polepole et al., 2017	FFPE tissue, extrapulmonary TB	Xpert MTB/RIF	Cartridge-based testing feasible and informative on paraffin-embedded material	Supports retrospective testing of archived blocks
Lo et al., 2024	FFPE tissue	Xpert MTB/RIF Ultra	Detects MTB in the majority of specimens positive by culture or reference PCR	Extends molecular yield to previously untestable archived specimens
Present case, 2026	Placenta (1), fresh; blood	Histopatho logy; ZN/Fite/G MS/Warthi n–Starry; CBNAAT; mycobacte rial blood culture	Caseating granulomatous villitis; all stains negative; CBNAAT positive, rifampicin sensitive; blood culture positive	Established diagnosis and susceptibility after all maternal airway and CSF specimens failed

Where molecular assays have been compared directly with staining on tissue, the difference is substantial. In 80 archival specimens with histology suggestive of tuberculosis, Xpert MTB/RIF identified 53.2% against 20.3% for Ziehl-Neelsen (p = 0.002) [12]**.** Comparable performance has been reported for CBNAAT testing of paraffin-embedded tissue in other series [13], and Xpert Ultra detects M. tuberculosis in the majority of the archived specimens positive by culture or reference PCR [14]**.** Two practical implications follow; first, fresh tissue is preferable to fixed tissue and should be requested at the time of delivery, and second, importantly for many cases in which tuberculosis is considered only retrospectively – the paraffin block retains a diagnostic value, so a stored placenta remains testable weeks or months later as well.

The placenta also carries specific weight in assessment of the neonate. Under the Cantwell criteria, demonstrated tuberculosis infection of the placenta or the maternal genital tract satisfies as one of the criteria for congenital tuberculosis, provided that the tuberculous lesions are also proven in the infant [15]**.** A positive placental molecular result therefore does more than just confirming the maternal diagnosis; it should trigger immediate mycobacteriological evaluation of the neonate. Gastric or endotracheal aspirate for NAAT, culture and hepatic imaging should inform decisions regarding empirical neonatal therapy. In the series cited above, 1/3rd of surviving infants born to mothers with molecularly confirmed placental tuberculosis developed the congenital disease [8]**.** A contemporaneous case of miliary tuberculosis presenting as ARDS in pregnancy lacked neuro-axial and placental involvement and microbiological confirmation [16]; tuberculosis in pregnancy is independently associated with preterm birth and perinatal death [17], which is a strong argument for treating a positive placenta as an actionable pediatric finding rather than a retrospective curiosity. In our patient, this opportunity was lost; the neonate underwent neither mycobacteriological evaluation nor a post mortem examination, and congenital tuberculosis therefore remained unproven. Endotracheal aspirate for CBNAAT should also have been sent.

Several limitations merit acknowledgement – tuberculous meningitis was classified as probable than definite, and cerebrospinal fluid sampling was undertaken during corticosteroid exposure, which attenuates pleocytosis. Bronchoscopic sampling was never undertaken, including during the interval of clinical recovery when it was feasible. As a single case, the report is hypothesis generating, and the accompanying review is focused rather than systematic, and the studies summarised in Table 2 differ in design, specimen handling and reference standard so the yields shown are not directly poolable. Prospective evaluation of molecular placental testing against maternal and neonatal outcomes is warranted.

The practical message is nonetheless simple – where tuberculosis is suspected in a pregnant woman, the placenta should be sent fresh, divided and submitted for histopathology, acid-fast and fungal staining, mycobacterial culture and CBNAAT, not histopathology alone. In our patient this single request converted a clinically suspected but microbiologically unproven illness into a confirmed susceptibility characterised diagnosis at no procedural risk to a critically ill mother.

## CRediT authorship contribution statement

**Bhvika Zutshi:** Writing – review & editing, Writing – original draft, Visualization, Formal analysis, Conceptualization. **Sandeep Garg:** Writing – review & editing, Validation, Supervision, Project administration. **Praveen Bharti:** Writing – review & editing, Investigation. **Amit Kumar:** Writing – review & editing, Methodology, Investigation, Data curation. **Annesha Chakraborty:** Writing – review & editing, Resources, Methodology, Investigation, Formal analysis. **Priyadarshi Meena:** Writing – review & editing, Investigation, Data curation.

## Patient consent and ethical considerations

Written informed consent for the publication of patient’s clinical details, placental and neonatal details and accompanying images was obtained from the patient, in a language she understands. Reporting conforms to the CARE (CAse REport) guidelines. Institutional ethical approval for publication of de-identified case material was obtained in accordance with local policy.

## Consent for publication

Written informed consent for publication of clinical details and images was obtained from the patient.

## Funding

This work received no specific grant from any funding agency in the public, commercial or not-for-profit sectors.

## Declaration of Competing Interest

The authors declare that they have no known competing financial interests or personal relationships that could have appeared to influence the work reported in this paper.
